# On the Effects of Poisoning with Arsenic

**Published:** 1815-12

**Authors:** W. J. Crowfoot

**Affiliations:** Member of the Royal College of Surgeons.


THE LONDON
### Medical and Physical Journal.

6 OF VOL. XXXIV.] DECEMBER, 1815. [NO. 202.

"For many fortunate discoveries in medicine, and for the detection of nume-
"rous errors, the world is indebted to the rapid circulation of Monthly
"Journals; and there never existed any work to which the Faculty in
"EUROPE and AMERICA were under deeper obligations than to the
"Medical and Physical Journal of London, now forming a long, but an
"invaluable, series."—RUSH.

*For the London Medical and Physical Journal.*

### *On the Effects of Poisoning with Arsenic;* by W. J. CROWFOOT, Esq. Member of the Royal College of Surgeons.

IT has been observed, by an intelligent writer, "that medical opinions are valuable only when founded on incontrovertible facts, and that induction in this science should be made as much from experience and experiment, and as clearly and as faithfully, as in any other branch of natural philosophy." The subjoined case formed the subject of a criminal prosecution; and the fact, so far as this solitary fact goes, appears to confirm the doctrine of Mr. Brodie, that death from the poison of arsenic, when produced shortly after its exhibition, does not arise from inflammation.

Robert Sparkes, a child about six years old, on the morning of the third of July, 1815, partook, with five other children, of a small plum-cake which had been given them as a present. Robert S. ate more heartily of it than the others. Very soon after, they were all taken extremely ill. Those that were old enough to describe their feelings complained of sickness, and pain across the stomach, and a general sense of coldness. Robert S. the subject of this case, was colder than the rest. Emetics were administered to each from the reasonable supposition that poison had been swallowed. These produced the desired effect upon all except this child. He became worse, was seized with convulsions, and in a strong convulsive fit expired about five hours from his having eaten the cake. A part of the cake remaining, it was brought to me by Mr. Dashwood (the gentleman who attended) with a request to analyse its contents, and the following was the mode I adopted.

To about six or eight ounces of distilled water was added a small portion of the cake; this, being suffered to boil for five minutes, was then filtered.

 Exp. 1.—

Exp. 1.—To a portion of the clear liquor was added the minutest drop of pure ammonia, a stick of nitrate of silver was then brought into contact with the fluid, and immediately a copious bright yellow coloured precipitate took place, thereby proving arsenic to be present.

Exp. 2.—To a like quantity of the filtered liquor was added a drop of a weak solution of carbonate of potash, a piece of dry sulphate of copper was then held in contact with the fluid, and very shortly a precipitate of a green colour (known by the name of Scheele's green) took place, which likewise indicated the presence of arsenic.

Exp. 3.—With another portion of the fluid I tried the sulphuret of ammonia, which also confirmed the preceding experiments. In addition to which, the same trials were made with a solution of arsenic itself, in the proportion of two grains to eight ounces of distilled water, and not a shade of difference could be observed. Having thus satisfied myself of the presence of arsenic in the cake, it was next necessary to examine the contents of the stomach of the deceased child. Accordingly, on the following morning, the body was opened by Mr. Dashwood in my presence; and, the orifices of the stomach being carefully secured, the stomach itself was removed: its contents (which consisted of about four or five ounces of liquid, with some small portions of the cake floating therein,) being subjected to the same tests as those before described, similar results were produced, and the fact verified beyond all question; however, the common proof was not neglected of enclosing a portion of the dried contents of the stomach between two copper plates, which gave evidently, though not strongly, the usual silvery appearance, and added to the already satisfactory evidence of the presence of arsenic. From the whole, therefore, though every experiment produced conviction of the fact, it may be useful to state that the weakest proof was that by the action of heat, and the strongest and most decisive by the admirable test of Mr. Hume.

It will be proper and necessary to observe, that, upon laying open the stomach, an appearance of inflammation was remarked about the lower orifice, so slight as to excite our admiration; as we had expected, from the proofs already established of the exhibition of the poison, that its destructive activity would have been decidedly manifest upon the coats of that viscus. This is an inquiry of considerable importance, and I believe the prevailing opinion upon the subject is, that the inflammation occasioned by the actual contact of arsenic with the internal coat of the stomach is such as to be the immediate cause of death! In the case recited, are are we warranted in such a conclusion? If we reason from analogy, perhaps the experiments so ably conducted by Mr. Brodie, will tend to settle our opinion upon this subject. He says, "When an animal is killed by arsenic taken internally, the stomach is found bearing marks of inflammation, and it is a very general opinion, 1st, that this inflammation is the cause of death; 2d, that it is the consequence of the actual contact of the arsenic with the internal coat of the stomach; but, in several cases, I have found the inflammation of the stomach so slight, that, on a superficial examination, it might have been overlooked; and, in most of my experiments with this poison, death has taken place in too short a time for it to be considered as the result of inflammation." He further observes, "the symptoms produced by arsenic may be referred to the influence of the poison on the nervous system, the heart, and the alimentary canal. As, of these, the two former only are concerned in those functions which are directly necessary to life, and as the alimentary canal is often affected only in a slight degree, we must consider the affection of the heart and nervous system as being the immediate cause of death.

*Beccles;*
*October* 10, 1815.

---

*For the London Medical and Physical Journal.*

*Case of Peritonitis;* by R. N. Starr, Esq. Surgeon.

